# Combination of *Pelargonium sidoides* and *Coptis chinensis* root inhibits nuclear factor kappa B-mediated inflammatory response in vitro and in vivo

**DOI:** 10.1186/s12906-018-2088-x

**Published:** 2018-01-19

**Authors:** Sang Mi Park, Byung-Gu Min, Ji Yun Jung, Kyung Hwan Jegal, Chul Won Lee, Kwang Youn Kim, Young Woo Kim, Youn-Woong Choi, Il Je Cho, Sae Kwang Ku, Sang Chan Kim

**Affiliations:** 1 College of Korean Medicine, Daegu Haany University, Gyeongsan, 38610 Republic of Korea; 2 Chungnam National University, Daejeon, 34134 Republic of Korea; 3 Korea United Pharm Inc., Seoul, 06116 Republic of Korea

**Keywords:** *Pelargonium sidoides*, *Coptis chinensis* root, Inflammation, Nuclear factor kappa B, Paw edema, Histopathology

## Abstract

**Background:**

*Pelargonium sidoides* (PS) and *Coptis chinensis* root (CR) have traditionally been used to treat various diseases, including respiratory and gastrointestinal infections, dysmenorrhea, and hepatic disorders. The present study was conducted to evaluate the anti-inflammatory effects of a combination of PS and CR in vitro and in vivo.

**Methods:**

The in vitro effects of PS + CR on the induction of inflammation-related proteins were evaluated in lipopolysaccharide (LPS)-stimulated RAW 264.7 cells. The levels of nitric oxide (NO) and of inflammatory cytokines and prostaglandin E_2_ (PGE_2_) were measured using the Griess reagent and enzyme-linked immunosorbent assay (ELISA) methods, respectively. The expression of inflammation-related proteins was confirmed by Western blot. Additionally, the effects of PS + CR on paw edema volume, skin thickness, and numbers of infiltrated inflammatory cells, mast cells, COX-2-, iNOS-, and TNF-α-immunoreactive cells in *dorsum* and *ventrum pedis* skin were evaluated in a rat model of carrageenan (CA)-induced paw edema.

**Results:**

PS + CR significantly reduced production of NO, PGE_2_ and three pro-inflammatory cytokines (tumor necrosis factor-α (TNF-α), interleukin (IL)-1β, and IL-6) and also decreased levels of inducible nitric oxide synthase (iNOS) and cyclooxygenase-2 (COX-2). Treatment with PS + CR significantly reduced the protein expression levels of LPS-stimulated nuclear factor kappa B (NF-κB) and phosphorylated inhibitor of NF-κB (p-I-κBα). Additionally, PS + CR significantly inhibited the increases in paw swelling, skin thickness, infiltrated inflammatory cells, mast cell degranulation, COX-2-, iNOS-, and TNF-α-immunoreactive cells in the rat model of CA-induced acute edematous paw.

**Conclusions:**

These results demonstrate that PS + CR exhibits anti-inflammatory properties through decreasing the production of pro-inflammatory mediators (NO, PGE_2_, TNF-α, IL-1β, and IL-6), suppressing NF-κB signaling in LPS-induced RAW 264.7 cells. Additionally, the results of the CA-induced rat paw edema assay revealed an anti-edema effect of PS + CR. Furthermore, it is suggested that PS + CR also inhibits acute edematous inflammation by suppressing mast cell degranulation and inflammatory mediators (COX-2, iNOS, and TNF-α). Thus, PS + CR may be a potential candidate for the treatment of various inflammatory diseases, and it may also contribute to a better understanding of the molecular mechanisms underlying inflammatory response regulation.

## Background

Inflammation is an important immune response for defending against harmful stimuli, such as pathogenic bacteria, viruses, and fungi [1, 2]. Once infected with a pathogen, the skin and mucosal surfaces serve as primary physical barriers in the immune response that maintains homeostasis [3]. However, excessive inflammation is a key player in the development of multiple diseases, including cancer, diabetes, inflammatory bowel diseases, and cardiovascular diseases [4]. Inflammation is tightly regulated by signals that initiate, maintain, or inhibit the inflammatory process [5, 6]. Inflammation has diverse triggers, including infection, physical and chemical injuries, ischemia, and excessive immune response [3, 4].

Under inflammatory conditions, several enzymes, cytokines, and chemokines are secreted by macrophages as signaling molecules to resolve abnormal conditions [1, 7]. The model most commonly used to investigate inflammation involves activating macrophage cells with lipopolysaccharides (LPS) to trigger the abnormal generation of nitric oxide (NO), prostaglandin E_2_ (PGE_2_), tumor necrosis factor α (TNF-α), and interleukin (IL)-1β [8, 9]. Recently, there have been several reports regarding the use of traditional medicine or phytomedicine to treat various diseases related to inflammation, such as allergies, asthma, eczema, rheumatoid arthritis, irritable bowel syndrome, and cancer [10, 11].

In South Africa, the polyphenol-rich herbal roots of the plant *Pelargonium sidoides* are traditionally used to treat respiratory and gastrointestinal infections, dysmenorrhea, and hepatic disorders [12, 13]**.** They have also been used in tuberculosis, and their medicinal properties include antibacterial, antifungal, antiviral, and immune modulatory activities [14]. Additionally, the dried rhizome of *Coptis chinensis* has been used to treat gastroenteric disorders, cardiovascular diseases, cancer, and liver injuries [15, 16]. Recent research has shown that these roots have pharmacological properties, such as anti-oxidant, anti-cancer, and anti-inflammatory activities [17–19].

It is expected that a combination of *P*. *sidoides* (PS) and *C. chinensis* root (CR) would have anti-inflammatory effects both in vitro and in vivo because it has traditionally been used to reduce fevers [3, 20]. However, the mechanisms underpinning the anti-inflammatory activities of the PS and CR mixture (PS + CR) have not yet been reported. Therefore, in this study, we evaluated the anti-inflammatory effects of PS + CR using LPS-stimulated RAW 264.7 cells as well as carrageenan (CA)-induced paw edema as an acute phase inflammation animal model.

## Methods

### Chemicals and reagents

The LPS (*E. coli* 026:B6), Griess reagent, 3-(4,5-dimethylthiazol)-2,5-diphenyltetrazolium bromide (MTT), epicatechin and berberine were obtained from Sigma (St. Louis, MO, USA). Enzyme linked immunesorbent assay (ELISA) kits for tumor necrosis factor-alpha (TNF-α), interleukin (IL)-6 and IL-1β were acquired from Pierce Endogen (Thermo Scientific, Waltham, MA, USA). The PGE_2_ ELISA kit was obtained from R&D Systems (Minneapolis, MN, USA). Anti-p-I-κBα, antibody and peroxidase-conjugated secondary antibody were purchased from Cell Signaling (Danvers, MA, USA). Anti-COX-2 and anti-iNOS antibodies were obtained from BD Biosciences (San Jose, CA, USA), while the anti-NF-κB, anti-I-κBα, anti-β-actin and anti-lamin A/C antibodies were purchased from Santa Cruz Biotechnology (Dallas, TX, USA). All other chemicals were purchased from the Sigma Chemical Co. (St. Louis, MO, USA).

### Preparation of *PS + CR* and determination of active compounds

*Pelargonium sidoides* extract and *Coptis chinensis* root extract powder were purchased from Sungil Bioex (Hwaseong, Republic of Korea) and identified by Dr. Byung Gu Min of the Korea United Pharm. Inc. in South Korea. The voucher specimens (KUP TR001 for *PS*; KUP M003 for *CR*) are deposited in the Korea United Pharm. Inc.. Briefly, *P. sidoides* extract is an important aqueous-ethanolic (11% m/m) extract with a yield of 8–10%. In addition, *C. chinensis* roots (1 kg) were extracted with 7 L of boiling distilled water for 3 h, and filtered through a filter paper (Hyundai Micro No. 20). The supernatant was filtered through a 0.2 μm filter (Nalgene, New York, USA), and the filtrate was then lyophilized. The yield of lyophilized *C. chinensis* root extract was 11.2%. Furthermore, *P. sidoides* extract was treated with *C. chinensis* root extract powder (2:1). For this, extract (10 g) was dissolved in distilled water (250 ml), *Coptis chinensis* powder (5 g) added and the mixture stirred for 45 min at 25 °C. The sample was then lyophilized and kept at − 20 °C until use. The lyophilized powder was dissolved in sterile water prior to use.

An ultra-performance liquid chromatography (UPLC) analysis was performed using the Waters ACQUITY UPLC system (Waters Corporation, Milford, MA, USA) with a photodiode array detector (PDA), BEH C_18_ HPLC column (1.7 μm, 2.1 mm × 100 mm), and Empower software. Samples were extracted with an 8210R–DHT ultrasonic cleaner (Branson Company) using methanol (JT BAKER for the HPLC), acetonitrile (JT-BAKER for the HPLC), dimethylsulfoxide (DMSO; Sigma for the HPLC), and tertiary distilled water. The standard preparations for this experiment were from WAKO Co. (Japan). In the PDA wavelength analysis, epicatechin and berberine were detected at 230 nm and 345 nm, respectively. The mobile phase was a mixed liquid of water and acetonitrile containing 0.1% formic acid. Samples were injected in 2 μl aliquots, and the flow rate was 0.4 ml/min.

### Cell culture and cell viability test

The RAW 264.7 cell line was obtained from the American Type Culture Collection (ATCC, Manassas, VA, USA) and maintained in Dulbecco’s modified Eagle’s medium (DMEM, Hyclone, Logan, UT, USA) containing 10% fetal bovine serum (FBS, Hyclone) at 37 °C in a humidified atmosphere with 5% CO_2_. For all experiments, LPS was used at a final concentration of 1 μg/ml after serum starvation for 12 h. Various concentrations of PS + CR were added to the culture medium 1 h prior to the addition of LPS, and cells were treated for 24 h. After treatment, viable cells were stained with 3-(4,5-dimethylthiazol)-2,5-diphenyltetrazolium bromide (MTT) and dissolved in DMSO. Absorbance was measured at 540 nm using a Tecan Infinite M200 PRO ELISA microplate reader (Tecan Group Ltd., Männedorf, Switzerland). Cell viability was defined relative to untreated controls by the equation: viability (% control) = 100 × (absorbance of treated sample/absorbance of control).

### Determination of NO, PGE_2_, and cytokines

Concentrations of NO were monitored by measuring the nitrite content in the culture supernatant after mixing the culture media with Griess reagent (1% sulphanilamide, 0.1% *N*-1-naphthylenediamine dihydrochloride, and 2.5% phosphoric acid). The absorbance was measured at 540 nm after 10 min of incubation. The levels of PGE_2_ and pro-inflammatory cytokines TNF-α, IL-1β, and IL-6 were assessed by enzyme-linked immunosorbent assay (ELISA) using anti-mouse PGE_2_, TNF-α, IL-1β, or IL-6 antibodies and a biotinylated secondary antibody according to the manufacturer’s instructions.

### Western blot analysis

Cell extracts were prepared by lysing cells in RIPA Lysis Buffer (Thermo Fisher Scientific, Waltham, MA, USA). The nuclear fractions for the NF-κB analysis were set up using a commercial kit (Chemicon International, Inc., Billerica, MA, USA). Equal amounts of proteins were separated using sodium dodecyl sulfate polyacrylamide gel electrophoresis (SDS-PAGE) and transferred to a nitrocellulose membrane (Amersham Life Science, Arlington Heights, IL, USA). After blocking with TBS-T buffer [20 mM Tris (pH 7.4), 150 mM NaCl, 0.1% Tween 20] containing 5% skim milk, the membranes were incubated with primary and secondary antibodies. The membranes were washed with TBS-T buffer and visualized with ECL Western blotting detection reagents (Amersham).

### Carrageenan-induced paw edema

Sprague–Dawley rats (6-week-old males, 140–160 g) were provided by Samtako Bio (Osan, Korea), allowed to acclimate to their new environment for 1 week, and maintained in a clean room at the animal laboratory. The animals were caged with a supply of filtered pathogen-free air, a temperature between 20 and 23 °C, a 12-h light:dark cycle, and a relative humidity of 50%; they were fed standard rat chow (Purina, Korea) and water ad libitum. The rats were divided into five groups: normal; CA; CA + dexamethasone (DEXA, 1 mg/kg) as a standard reference; CA + PS + CR (0.3 g/kg); and CA + PS + CR (1.0 g/kg). PS + CR and DEXA were dissolved in saline and orally administered to the control rats for 4 consecutive days. Paw edema was induced by injecting 0.1 ml of 1% *w*/*v* CA suspended in saline into the sub-plantar tissues of the right hind paw of each rat. The volume of the paw was measured with a Plethysmometer (LE 7500; LETICA Scientific Instruments, Barcelona, Spain) immediately prior to CA injection and then again 1, 2, 3, and 4 h after injection. After euthanasia with CO_2_, the paw samples were prepared.

### Immunohistochemistry

The separated *dorsum* and *ventrum pedis* skin from the hind paws were fixed in 10% neutral buffered formalin and then embedded in paraffin, sectioned, and stained with hematoxylin and eosin (HE) to determine general histopathological profiles. Next, changes in the immunoreactivities of cyclooxygenase-2 (COX-2), inducible nitric oxide synthase (iNOS), and TNF-α were observed with purified primary antibodies with avidin-biotin-peroxidase (ABC) and peroxidase substrate kits (Vector Labs, Burlingame, CA, USA). Briefly, the endogenous peroxidase activity was blocked by incubation in methanol and 0.3% H_2_O_2_ for 30 min, while non-specific binding of immunoglobulin was blocked with normal horse serum blocking solution for 1 h in a humidity chamber after heating (95–100 °C) for epitope retrieval in 10 mM citrate buffer (pH 6.0). The primary antisera were treated overnight at 4 °C in a humidity chamber and then incubated with biotinylated universal secondary antibody and ABC reagents for 1 h at room temperature in a humidity chamber. Finally, the sections were treated with a peroxidase substrate kit for 3 min at room temperature.

### Histomorphometry

To observe the changes induced by CA either with or without PS + CR treatment in greater detail, the thicknesses of the *dorsum* and *ventrum pedis* skin (from the epidermis to the dermis; keratin layers were excluded) were measured as μm/paw with an automated image analyzer (iSolution FL ver 9.1, IMT i-Solution Inc., Vancouver, Canada) under 40 X magnification microscopy (Model Eclipse 80i, Nikon, Tokyo, Japan). Using an automated image analyzer under 200 X magnification, we also counted the number of infiltrated inflammatory cells in the dermis as cells/mm^2^ of dermis. Additionally, the cells occupied by over 10% of COX-2 and iNOS immunoreactivities were regarded as positive. In the present study, the number of COX-2- and iNOS-positive cells were separately calculated in the epithelial lining (cells/100 epithelial cells) and in the dermis (cells/mm^2^ of dermis) using a digital image analyzer. The histopathologist was blind to the group distribution when this analysis was carried out.

### Statistical analysis

The experimental results are presented as the mean ± standard deviation (S.D.) of experiments repeated at least three times. In each treatment group, statistical significance was compared and verified using one-way analysis of variance (ANOVA) or Student’s *t*-test (*p* < 0.05 or *p* < 0.01). Additionally, the percentage point (pp) changes between normal and CA groups were calculated to monitor the severities of acute inflammation induced in this study; to assess efficacy, the pp. changes between CA and PS + CR- or DEXA-treated skin were also calculated as follows:

Percentage Point Changes compared with Normal (%) = ((Data of CA – Data of Normal)/Data of Normal) × 100.

Percentage Point Changes compared with CA (%) = ((Data of PS + CR-treated rats – Data of CA)/Data of CA) × 100.

## Results

### UPLC analysis of PS + CR

**Fig. 1:**
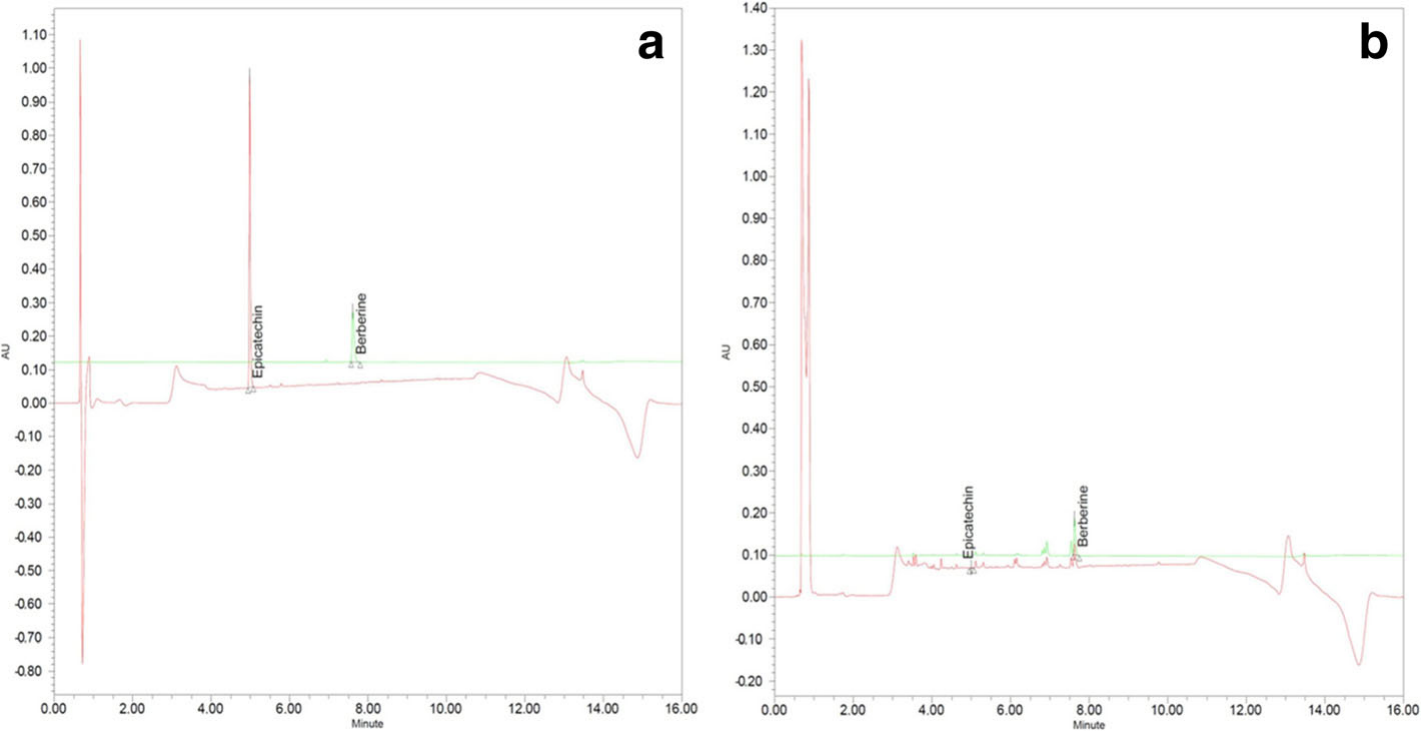
Analysis of compounds in a mixture of *Pelargonium sidoides* and *Coptis chinensis* root (PS + CR) by ultra-performance liquid chromatography (UPLC). UPLC chromatograms of standard compounds; epicatechin: 230 nm, berberine: 345 nm. **a** standard compounds. **b** PS + CR extracts

To evaluate the quantitative UPLC analysis of PS + CR, we used two standards for quality control based on the Korean Pharmacopoeia and the Korean Food and Drug Administration. The contents of the two compounds were calculated from the calibration curves of the standards (Fig. 1). All standard peaks were identified within 4–8 min of retention time, and the following amounts of compound were detected: 0.149 ± 0.004 ppm for epicatechin and 37.29 ± 0.11 ppm for berberine.

### Effects of PS + CR on NO production and iNOS protein expression

**Fig. 2:**
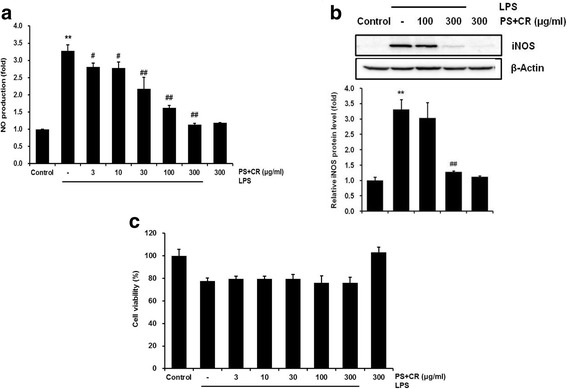
Inhibition of nitric oxide (NO) production and inducible nitric oxide synthase (iNOS) expression by PS + CR. RAW 264.7 cells were treated with 3–300 μg/ml PS + CR for 1 h prior to the addition of lipopolysaccharide (LPS) (1 μg/ml) and further incubated for 24 h. **a** Effects of PS + CR on NO production. Secreted nitrite concentrations in the culture medium were assayed using the Griess reagent method. **b** Effects of PS + CR on iNOS protein expression. Equal amounts of total protein (50 μg) were separated by SDS-PAGE. β-Actin was used as a loading control, and the bar chart shows the quantitative evaluation of iNOS bands by densitometry. (c) Effects of PS + CR plus LPS on cell viability. Cell viability was measured by 3-(4,5-dimethylthiazol)-2,5-diphenyltetrazolium bromide (MTT) assay after 24 h of incubation. Values represent the mean ± S.D. of three independent experiments (significant compared to the control, ^**^p < 0.01; significant compared to LPS alone, ^#^*p* < 0.05 or ^##^*p* < 0.01)

To confirm the anti-inflammatory effects of PS + CR, we investigated LPS-induced NO production in RAW 264.7 cells. The cells were treated with various concentrations (3–300 μg/ml) of PS + CR for 1 h, followed by continuous incubation with LPS (1 μg/ml) for 24 h; NO production was then measured using a Griess reagent. Treatment with 30–300 μg/ml PS + CR significantly inhibited the increased NO production induced by LPS (Fig. 2a, *p* < 0.01). To evaluate the possibility that inhibition of NO production by PS+CR was associated with modulated expression of the iNOS protein as a key enzyme responsible for NO synthesis, we performed Western blot analysis. As expected, LPS significantly increased iNOS protein expression; however, pretreatment with PS+CR (300 μg/ml) significantly inhibited the increased iNOS expression induced by LPS (Fig. 2b, *p* < 0.01). Furthermore, cell viability was not affected by co-treatment with LPS and PS+CR (Fig. 2c). These results demonstrated that the inhibitory effect of PS+CR on LPS-stimulated NO production was not due to cytotoxic effects.

### Effects of PS + CR on PGE_2_ production and COX-2 protein expression

**Fig. 3:**
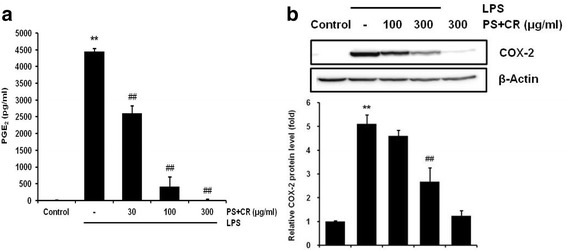
Inhibition of the LPS-induced prostaglandin E_2_ (PGE_2_) secretion and cyclooxygenase-2 (COX-2) expression by PS + CR. RAW 264.7 cells were treated with 30–300 μg/ml of PS + CR for 1 h prior to the addition of LPS (1 μg/ml) and further incubated for 24 h. **a** Effects of PS + CR on the levels of PGE_2_. PGE_2_ concentrations in the culture medium were assayed using the PGE_2_ ELISA kit as detailed in the Methods section. **b** Effects of PS + CR on COX-2 protein expression. Equal amounts of total protein (50 μg) were separated by SDS-PAGE. β-Actin was used as a loading control, and the bar chart shows the quantitative evaluation of COX-2 bands by densitometry. Values represent the mean ± S.D. of three independent experiments (significant compared to the control, ^**^p < 0.01; significant compared to LPS alone, ^##^*p* < 0.01)

To investigate whether PS + CR inhibits production of PGE_2_ and expression of COX-2 as a potent mediator of inflammation [21], we utilized ELISA and Western blot analysis, respectively. Treatment with LPS significantly increased production of PGE_2_; however, treatment with PS + CR at concentrations of 30–300 μg/ml significantly suppressed this increased production (Fig. 3a, *p* < 0.01). Next, we evaluated the effects of PS+CR on COX-2 and found that PS+CR (300 μg/ml) significantly inhibited LPS-enhanced COX-2 expression (Fig. 3b, *p* < 0.01). These results suggest that PS+CR may inhibit PGE_2_ synthesis by regulating expression of COX-2.

### Effects of PS + CR on production of pro-inflammatory cytokines

**Fig. 4:**
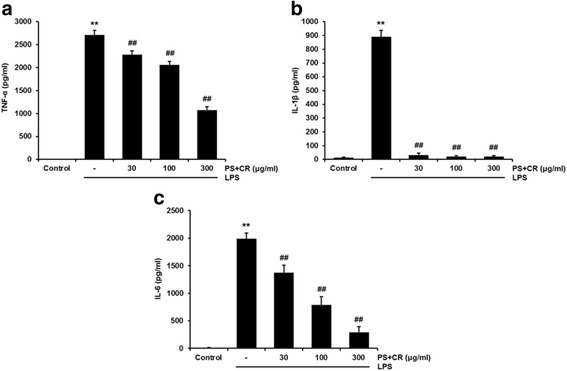
Inhibition of the LPS-induced secretion of pro-inflammatory cytokines by PS + CR. RAW 264.7 cells were treated with 30–300 μg/ml of PS + CR for 1 h prior to the addition of LPS (1 μg/ml) and further incubated for 24 h. Levels of TNF-α (**a**) IL-1β (**b**) and IL-6 (**c**). As detailed in the Methods section, the concentrations of cytokines in the culture medium were assayed using a kit. Values represent the mean ± S.D. of three independent experiments (significant compared to the control, ^**^p < 0.01; significant compared to LPS alone, ^##^*p* < 0.01)

We used ELISAs to evaluate whether PS + CR reduces the production of pro-inflammatory cytokines TNF-α, IL-1β, and IL-6. Treatment with LPS significantly increased production of TNF-α, IL-1β, and IL-6, but these increases were significantly reduced by treatment with 30–300 μg/ml PS + CR (Fig. 4a-c, *p* < 0.01).

### Effects of PS + CR on NF-κB signaling pathway

**Fig. 5:**
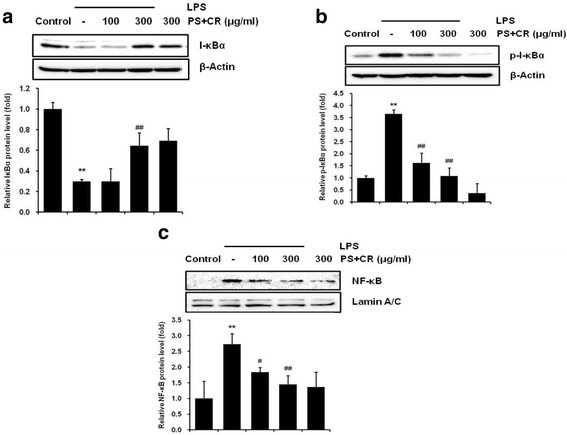
Inhibition of LPS-induced nuclear factor-kappa B (NF-κB) protein expression by PS + CR. (**a**, **b**) Levels of I-κBα and phospho-I-κBα. RAW 264.7 cells were treated with 100 and 300 μg/ml of PS + CR for 1 h prior to the addition of lipopolysaccharide (LPS) (1 μg/ml) and further incubated for 30 min. Cytosol fraction proteins were separated by SDS-PAGE. β-Actin was used as a loading control, and the bar chart shows the quantitative evaluation of I-κBα and phospho-I-κBα bands by densitometry. (c) The levels of NF-κB. RAW 264.7 cells were treated with 100 and 300 μg/ml of PS + CR for 1 h prior to the addition of LPS (1 μg/ml) and further incubated for 1 h. Nuclear fraction proteins were separated by SDS-PAGE. Lamin A/C was used as a loading control, and the bar chart shows the quantitative evaluation of NF-κB bands by densitometry. Values represent the mean ± S.D. of three independent experiments (significant compared to the control; ^**^p < 0.01, significant compared to LPS alone, ^#^*p* < 0.05 or ^##^*p* < 0.01)

NF-κB is a key transcription factor with important roles in a wide range of biological effects, including inflammation [22]. To examine whether PS + CR regulates NF-κB nuclear translocation as well as I-κBα phosphorylation and degradation, we performed Western blot analysis. As expected, LPS treatment increased phosphorylated I-κBα (p-I-κBα) protein expression and decreased total I-κBα protein expression (Fig. 5a, b). However, pretreatment with 300 μg/ml PS + CR significantly decreased the phosphorylation of I-κBα and blocked the LPS-induced degradation of I-κBα (Fig. 5a, b; *p* < 0.01), suggesting that PS + CR may regulate phosphorylation of I-κBα and I-κBα expression. Furthermore, the accumulation of NF-κB in nuclear fractions following LPS treatment was significantly and dose-dependently reduced by PS + CR pretreatment (Fig. 5c, *p* < 0.05 for 100 μg/ml; *p* < 0.01 for 300 μg/ml). These results indicate that PS + CR inhibits NF-κB activation by inhibiting I-κBα degradation and the nuclear translocation of NF-κB.

### Effects of PS + CR on CA-induced paw edema

**Fig. 6:**
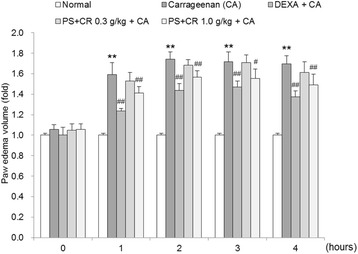
Inhibition of carrageenan (CA)-induced paw edema by PS + CR. PS + CR was orally administered to rats at 0.3 or 1.0 g/kg/day for four days prior to the induction of paw edema. Paw edema was induced by subcutaneous injection of a 1% solution of CA dissolved in saline (0.1 ml per animal) into the right hind paw of each rat. The swelling volume of the paw was measured up to 4 h after the CA injection at intervals of 1 h using a plethysmometer. Dexamethasone (DEXA) (1 mg/kg, p.o.) was used as a positive control. Values represent the mean ± S.D. of five animals (significant compared to the normal, ^**^p < 0.01; significant compared to CA alone, ^#^*p* < 0.05 or ^##^*p* < 0.01)

To determine the anti-inflammatory effects of PS + CR, we used a CA-induced paw edema method as an in vivo model of acute inflammation. The inhibition of paw edema formation by PS + CR was calculated by comparing the hind paw volumes of rats treated with this combination with those of CA-treated rats. Additionally, DEXA (1 mg/kg) was used as a positive control. As shown in Fig. 6, DEXA and PS + CR (0.3 and 1.0 g/kg) significantly inhibited paw edema (*p* < 0.01 for DEXA, *p* < 0.05 or *p* < 0.01 for 1.0 g/kg PS + CR).

### Histopathological evaluation of PS + CR in CA-induced acute inflammation

**Fig. 7:**
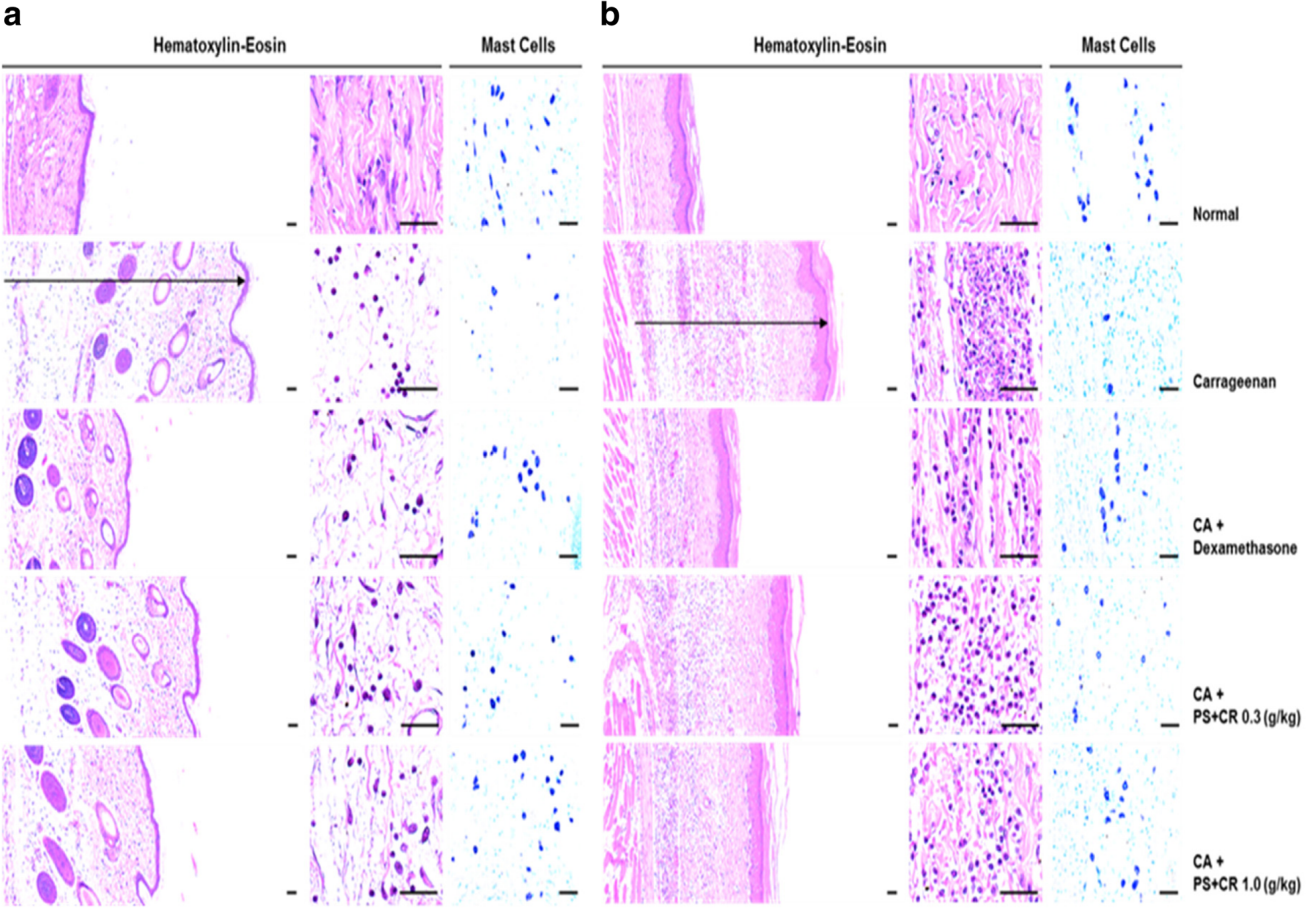
Inhibition of increased paw skin thickness and infiltrating inflammatory cells by PS + CR. Histological profiles and infiltrated inflammatory cells in the *dorsum pedis* skin (**a**) and *ventrum pedis* skin (**b**). CA treatment markedly increased skin thickness (arrow) and inflammatory cell infiltration due to edematous changes. Scale bars = 60 μm

**Fig. 8:**
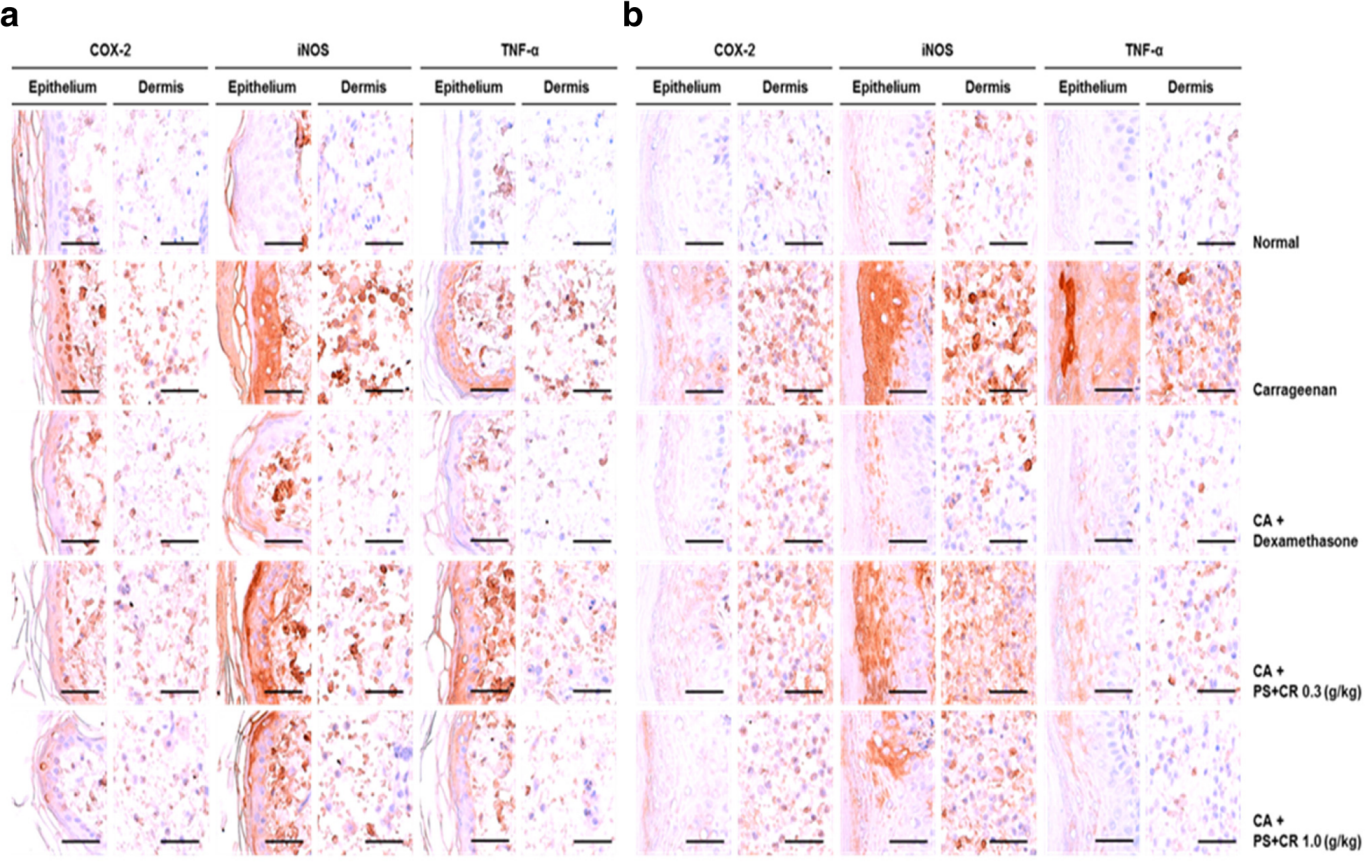
Representative immunohistochemical profiles of COX-2, iNOS, and TNF-α in paw skin. Marked increases of COX-2, iNOS, and TNF-α-positive cells were detected in the epithelium and dermis of the *dorsum* (**a**) and *ventrum pedis* skin tissues (**b**) in CA-treated rats compared with normal rats. Scale bars = 60 μm

The histological profiles, including mast cell stains, of the effects of PS + CR treatment on the *dorsum* and *ventrum pedis* skin are shown in Fig. 7. Additionally, the representative immunohistochemical profiles of the epithelial and dermal COX-2, iNOS, and TNF-α immunolabeled cells in the *dorsum* and *ventrum pedis* skin tissues are shown in Fig. 8. The histomorphometrical measurements of the *dorsum pedis* and *ventrum pedis* skin are also listed in Tables 1 and 2, respectively.

**Table 1:** Changes on the histomorphometrical analysis of hind paw skins

Regions Index Groups	Dorsum pedis skin		Ventrum pedis skin	
	Thickness (epidermis to dermis; μm)	Infiltrated inflammatory cells (cells/mm^2^ of dermis)	Thickness (epidermis to dermis; μm)	Infiltrated inflammatory cells (cells/mm^2^ of dermis)
Normal	554.49 ± 45.78	33.20 ± 6.98	367.00 ± 47.70	38.60 ± 5.81
Carrageenan (CA)	1374.91 ± 133.42^a^	300.80 ± 48.15^a^	1103.50 ± 163.77^d^	1899.20 ± 240.07^d^
CA + Dexamethasone	705.25 ± 69.63^bc^	67.60 ± 19.51^c^	507.85 ± 70.23^ef^	552.80 ± 111.52^df^
CA + PS + CR 0.3 (g/kg)	1013.45 ± 89.90^ac^	197.60 ± 20.72^ac^	856.48 ± 83.57^df^	1167.60 ± 213.76^df^
CA + PS + CR 1.0 (g/kg)	885.66 ± 85.71^ac^	129.00 ± 41.48^ac^	605.99 ± 42.23^df^	808.20 ± 178.40^df^

Values are expressed as mean ± S.D. of five rat hind paws

^a^*p* < 0.01 and ^b^*p* < 0.05 as compared with normal by LSD test. ^c^*p* < 0.01 as compared with CA by LSD test

^d^*p* < 0.01 and ^e^ *p* < 0.05 as compared with normal by MW test. ^f^*p* < 0.01 as compared with CA by MW test

**Table 2:** Changes on the mast cells, COX-2-, iNOS- and TNF-α-immunoreactive cells in the hind paw skins

Regions/Cells	Normal Carrageenan (CA)		CA + Dexamethasone	CA + PS + CR 0.3 (g/kg)	CA + PS + CR 1.0 (g/kg)
*Dorsum pedis* skin					
Epidermis (cells/100 epithelial cells)					
COX-2 **+** cells	3.60 ± 2.61	77.60 ± 5.94^d^	27.80 ± 10.57^de^	59.60 ± 9.71^df^	36.00 ± 6.52^de^
iNOS + cells	4.40 ± 2.30	86.80 ± 5.81^a^	35.00 ± 7.52^ac^	73.80 ± 4.32^ac^	56.40 ± 8.41^ac^
TNF-α + cells	1.00 ± 0.71	87.20 ± 6.06^a^	11.40 ± 4.10^bc^	70.80 ± 7.46^ac^	41.40 ± 7.40^ac^
Dermis (cells/mm^2^ of dermis)					
Mast cells	90.40 ± 15.63	22.00 ± 7.52^a^	74.20 ± 4.44^bc^	48.00 ± 13.36^ac^	64.20 ± 13.59^ac^
COX-2 **+** cells	13.20 ± 6.57	160.00 ± 31.34^d^	38.80 ± 10.55^de^	103.80 ± 9.86^de^	56.20 ± 18.39^de^
iNOS + cells	18.60 ± 2.41	379.60 ± 81.04^d^	78.80 ± 12.13^de^	216.40 ± 71.14^df^	109.00 ± 54.16^de^
TNF-α + cells	10.20 ± 1.92	175.80 ± 21.31^a^	47.60 ± 12.93^ac^	112.20 ± 25.41^ac^	72.20 ± 17.67^ac^
*Ventrum pedis* skin					
Epidermis (cells/100 epithelial cells)					
COX-2 **+** cells	3.80 ± 1.70	67.40 ± 4.51^a^	24.00 ± 7.91^ac^	53.00 ± 7.18^ac^	39.40 ± 4.67^ac^
iNOS + cells	13.40 ± 3.58	63.00 ± 6.52^a^	23.40 ± 6.19^bc^	47.40 ± 6.58^ac^	35.40 ± 5.13^ac^
TNF-α + cells	5.20 ± 2.39	82.00 ± 10.89^a^	33.00 ± 11.58^ac^	56.40 ± 6.23^ac^	40.40 ± 12.60^ac^
Dermis (cells/mm^2^ of dermis)					
Mast cells	81.60 ± 10.90	7.20 ± 3.35^a^	57.80 ± 8.01^ac^	29.20 ± 7.19^ac^	49.00 ± 10.72^ac^
COX-2 **+** cells	33.40 ± 11.61	823.00 ± 104.48^a^	146.20 ± 88.37^bc^	476.40 ± 98.22^ac^	309.40 ± 79.63^ac^
iNOS + cells	27.60 ± 10.62	727.00 ± 102.27^a^	145.80 ± 50.93^bc^	485.00 ± 88.26^ac^	338.00 ± 54.35^ac^
TNF-α + cells	19.20 ± 4.60	794.20 ± 110.87^d^	57.20 ± 17.48^de^	308.00 ± 56.57^de^	144.00 ± 40.35^de^

Values are expressed as mean ± S.D. of five rat hind paws

^a^*p* < 0.01 and ^b^*p* < 0.05 as compared with normal by LSD test. ^c^*p* < 0.01 as compared with CA by LSD test

^d^*p* < 0.01 as compared with normal by MW test. ^e^*p* < 0.01 and ^f^*p* < 0.05 as compared with CA by MW test

Marked and significant (*p* < 0.01) increases in the thickness of the *dorsum* and *ventrum pedis* skin resulted from CA-induced acute edematous inflammation in CA-treated rats compared with normal rat paw skin. The *dorsum* and *ventrum pedis* cutaneous regions in CA-treated rats also displayed significant (*p* < 0.01) degranulation-related decreases in mast cell numbers; increases in infiltrated inflammatory cells; and increased epithelial and dermal COX-2-, iNOS-, and TNF-α-positive cells compared to control rat paw skin. However, the CA-induced acute edematous inflammatory changes were significantly (*p* < 0.01 or *p* < 0.05) and dose-dependently inhibited by treatment with 0.3 and 1.0 g/kg PS + CR, as well as by treatment with DEXA. According to histopathological observations, PS + CR treatment (1.0 g/kg) had a somewhat less pronounced anti-inflammatory effect than DEXA treatment on CA-induced acute inflamed hind paws (Tables 1 and 2; Figs. 7 and 8).

The thicknesses of *dorsum pedis* and *ventrum pedis* skin in CA-treated rats were changed by 147.96 and 200.68 pp., respectively, compared with normal controls. The pp. changes in the thickness of *dorsum pedis* skin in rats treated with DEXA, 0.3 and 1.0 g/kg PS + CR were − 48.71, − 26.29, and − 35.58%, respectively, compared with CA-treated rats, and they were − 53.98, − 22.39, and − 45.08%, respectively, in *ventrum pedis* skin compared with CA-treated rats. Additionally, the numbers of infiltrated inflammatory cells in the *dorsum pedis* and *ventrum pedis* skin in CA-treated rats were increased by 806.02 and 4820.21 pp., respectively, compared with normal controls. In rats treated with DEXA, 0.3 and 1.0 g/kg PS + CR, the pp. changes in the numbers of infiltrated inflammatory cells were − 77.53, − 34.31, and − 57.11%, respectively, in *dorsum pedis* skin, and they were − 70.89, − 38.52, and − 57.45%, respectively, in *ventrum pedis* skin compared with CA-treated rats.

The numbers of mast cells in the *dorsum pedis* and *ventrum pedis* skin in CA-treated rats were changed by − 75.66 and − 91.18 pp., respectively, compared with normal controls. In rats treated with DEXA, 0.3 and 1.0 g/kg PS + CR, the pp. changes in numbers of mast cells were 237.27, 118.18, and 191.82%, respectively, in *dorsum pedis* skin, and they were 702.78, 305.56, and 580.56%, respectively, in *ventrum pedis* skin compared with CA-treated rats. The numbers of epithelial COX-2-immunoreactive cells in the *dorsum pedis* and *ventrum pedis* skin in CA-treated rats were changed by 2055.56 and 1673.68 pp., respectively, compared with normal controls. In rats treated with DEXA, 0.3 and 1.0 g/kg PS + CR, the pp. changes in the numbers of COX-2-immunoreactive cells were − 64.18, − 23.20, and − 53.61%, respectively, in the epithelial *dorsum pedis* skin, and they were − 64.39, − 21.36 and − 41.54%, respectively, in epithelial *ventrum pedis* skin compared with CA-treated rats. Additionally, the numbers of dermal COX-2-immunoreactive cells in the *dorsum pedis* and *ventrum pedis* skin in CA-treated rats were changed by 1112.12 and 2364.07 pp., respectively, compared with normal controls. In rats treated with DEXA, 0.3 and 1.0 g/kg PS + CR, the pp. changes in numbers of COX-2-immunoreactive cells were − 75.75, − 35.13, and − 64.88%, respectively, in the dermal *dorsum pedis* skin, and they were − 82.24, − 42.11, and − 62.41%, respectively, in dermal *ventrum pedis* skin compared with CA-treated rats.

The numbers of epithelial iNOS-immunoreactive cells in the *dorsum pedis* and *ventrum pedis* skin in CA-treated rats were changed by 1872.73 and 370.15 pp., respectively, compared with normal controls. In rats treated with DEXA, 0.3 and 1.0 g/kg PS + CR, the pp. changes in numbers of epithelial iNOS-immunoreactive cells were − 59.68, − 14.98, and − 35.02%, respectively, in *dorsum pedis* skin, and they were − 62.86, − 24.76, and − 43.81%, respectively, in *ventrum pedis* skin compared with CA-treated rats. Moreover, the numbers of dermal iNOS-immunoreactive cells in the *dorsum pedis* and *ventrum pedis* skin in CA-treated rats were changed by 1940.86 and 2534.06 pp., respectively, compared with normal controls. In rats treated with DEXA, 0.3 and 1.0 g/kg PS + CR, the pp. changes in the numbers of dermal iNOS-immunoreactive cells were − 79.24, − 42.99, and − 71.29%, respectively, in *dorsum pedis* skin, and they were − 79.94, − 33.29, and − 53.51%, respectively, in *ventrum pedis* skin compared with CA-treated rats.

The numbers of epithelial TNF-α-immunoreactive cells in the *dorsum pedis* and *ventrum pedis* skin in CA-treated rats were changed by 8620.00 and 1476.92 pp., respectively, compared with normal controls. In rats treated with DEXA, 0.3 and 1.0 g/kg PS + CR, the pp. changes in the numbers of epithelial TNF-α-immunoreactive cells were − 86.93, − 18.81, and − 52.52%, respectively, in *dorsum pedis* skin, and they were − 59.76, − 31.22, and − 50.73%, respectively, in *ventrum pedis* skin compared with CA-treated rats. Furthermore, the numbers of dermal TNF-α-immunoreactive cells in the *dorsum pedis* and *ventrum pedis* skin in CA-treated rats were changed by 1623.53 and 4036.46 pp., respectively, compared with normal controls. In rats treated with DEXA, 0.3 and 1.0 g/kg PS + CR, the pp. changes in the numbers of dermal TNF-α-immunoreactive cells were − 72.92, − 36.18, and − 58.93%, respectively, in *dorsum pedis* skin, and they were − 92.80, − 61.22, and − 81.87%, respectively, in *ventrum pedis* skin compared with CA-treated rats.

## Discussion

In traditional medicine, different herbs are often mixed together to reduce toxicity and enhance efficacy [23]. Recent studies have reported that a mixture of *P*. *sidoides* and *C. chinensis* (PS + CR) has anti-oxidant, anti-cancer, and anti-inflammatory effects in diverse diseases [24, 25], however, the exact mechanisms of them remain to be elucidated. Here, we investigated the anti-inflammatory effects of PS + CR using two kinds of model: cells treated with LPS to mimic the inflammatory process to study the effects of drugs and their molecular mechanisms, and animals stimulated with carrageenan to induce an inflammatory response in the paw. In this study, we showed that PS + CR has strong anti-inflammatory effects in vitro and in vivo, and its action is mediated through regulating inflammation marker proteins as well as transcription factors in RAW 264.7 cells.

Several researchers have attempted to inhibit activated macrophages as a potential therapy for inflammatory disease [1, 26]. Many studies have shown that activating macrophages with bacterial endotoxins, such as LPS, primarily triggers the production of inflammatory mediators, including NO and PGE_2_, as well as the inducible enzymes iNOS and COX-2 [27–29]. iNOS is the primary regulator of NO production and a target of inflammation-associated tissue damage and attenuation of iNOS-mediated NO production [30]. COX-2 is known as the key enzyme in the synthesis of prostaglandins and is a mediator of inflammation, angiogenesis, and cancer progression [21, 31]. Furthermore, COX-2 has been used as a therapeutic target in inflammatory disease, with advantageous anti-inflammatory effects [32, 33]. Inhibiting iNOS and PGE_2_ synthesis can relieve chronic inflammation induced by LPS [3, 34, 35]. We observed that the significant increases in iNOS and COX-2 expression and the excessive production of NO and PGE_2_ induced by LPS were attenuated in the presence of PS + CR. These findings indicate that PS + CR may have a therapeutic effect by treating inflammatory symptoms and pathogenic pain.

Cytokines are related to the inflammation process in the human body. They are released by various inflammatory cells, including macrophages [23, 36]. TNF-α is the most important cytokine in inflammation and has a crucial function in cellular and tissue damage [37, 38]. IL-1β has an important role in the processes underpinning swelling, heating, and redness [39]. As a mediator of the inflammatory process, IL-6 is released primarily by macrophages [40]. In this study, PS + CR inhibited the LPS-induced production of the pro-inflammatory cytokines TNF-α, IL-1β, and IL-6 [37, 38]. Although PS + CR significantly inhibited three kinds of cytokine, it was more effective against the production of IL-1β than TNF-α or IL-6. Further investigation is needed to determine the different effects of PS + CR on the inhibition of inflammatory cytokines.

NF-κB is an important transcription factor of inflammatory molecules [22]. NF-κB is a functional transcription factor that regulates the expression of genes involved in programmed cell death, inflammation, and survival. Several studies have shown that NF-κB plays critical roles in regulating various pro-inflammatory enzymes and cytokines, such as iNOS, COX-2, TNF-α, IL-1β and IL-6 [24, 25]. Under normal conditions, NF-kB is located in the cytosol coupled with I-κBα, its inhibitory molecule. However, during an activation process such as inflammation, I-κBα is phosphorylated and degraded, and NF-κB moves to the nucleus by separating from I-κBα [41, 42]. In this study, LPS induced significant phosphorylation of I-κBα and nuclear translocation of NF-κB. Treatment with PS + CR significantly blocked the induction of the NF-κB signaling pathway by LPS. These results suggest that PS + CR may modulate the NF-κB signaling pathway in the process of inflammation.

Loosening of inflammatory cell infiltration and connective tissues was observed around CA-treated sites [43, 44]. In the present study, obvious increases in infiltrated inflammatory cells and increases in the skin thickness of both the *ventrum* and *dorsum pedis* were detected in CA-treated compared with control rat paws. However, these CA-induced acute inflammatory changes were significantly and dose-dependently reduced by treatment with two different doses of PS + CR (0.3 and 1.0 g/kg) and also by treatment with DEXA. These findings are considered direct evidence that PS + CR has favorable anti-inflammatory activities. The anti-inflammatory effects of PS + CR (1.0 g/kg) on the CA-induced edematous inflammatory skin changes were somewhat less pronounced than those of DEXA.

An obvious extension of mast cell degranulation in various dermal tissues has been observed in CA-induced acute inflammation, and inhibition of the mast cell degranulation has been used as an index of the efficacy of anti-inflammatory drugs [44–46]. In the present study, PS + CR markedly and dose-dependently inhibited mast cell degranulation and preserved the mast cell numbers in the dermis of CA-treated rats; mast cell degranulation was also significantly inhibited by treatment with DEXA in both *dorsum pedis* and *ventrum pedis* skin tissues. These results are considered direct evidence that PS + CR showed anti-inflammatory effects by at least partially controlling mast cell activation and degranulation.

Decreases in COX-2 immunoreactivity have been used as a valuable predictor of the favorable effects of test materials on inflammation [32, 33]. Additionally, iNOS is involved in the development of inflammation soon after CA administration, and NO produced by iNOS is related to the maintenance of the inflammatory response at later times [47]. TNF-α is involved in the pathogenesis of CA-induced inflammation [48]. In the present study, obvious increases in epithelial and dermal COX-2-, iNOS-, and TNF-α-immunoreactive cells were detected on the *dorsum pedis* and *ventrum pedis* skin in CA-treated rats compared to control rats. However, these increases in inflammatory mediator-immunolabeled cells were significantly and dose-dependently decreased by treatment with two different doses of PS + CR as well as by DEXA. Therefore, we concluded that, under these experimental conditions, PS + CR has an at least partial and favorable inhibitory effect on acute inflammation and that this effect involves inflammatory mediators (COX-2, iNOS, and TNF-α).

The results of chromatographic analysis showed that epicatechin and berberine are the two main markers of PS + CR. Treatment with epicatechin (0.1–1 mM) was found to inhibit the production of nitrite (a nitric oxide metabolite) in the rat β-cell line RINm5F and this compound was shown to block the IL-1β-induced expression of iNOS by inhibiting the nuclear localization of the p65 subunit of NF-κB [49]. In addition, Vasconcelos et al. [50] reported that treatment with epicatechin improved acute intestinal inflammatory disease. Furthermore, epicatechin exerted anti-inflammatory effects on diet-induced human C-reactive protein and NF-κB in vivo [51]. Berberine is an anti-inflammatory natural compound and is revealed to suppress hepatocyte inflammatory responses [52]. In addition, Guo et al. [53] suggested that berberine ameliorates obesity-associated liver and adipose tissue inflammation without altering AMPK phosphorylation. Furthermore, berberine suppresses the expression of acute phase proteins as well as pro-inflammatory cytokines, such as iNOS, COX-2, C-reaction protein, IL-1β, IL-6, and TNF-α and it inhibits inflammation via diverse mechanisms, such as the AMPK, NF-κB, and MAPK signaling pathways [54–56]. Therefore, we suggest that the anti-inflammatory properties of PS + CR on LPS-stimulated RAW 264.7 cells and CA-induced rat paw edema are likely due to epicatechin and berberine.

## Conclusions

Our results demonstrate that PS + CR exhibits anti-inflammatory properties by decreasing the production of pro-inflammatory mediators (NO, PGE_2_, TNF-α, IL-1β, and IL-6), suppressing NF-κB signaling in LPS-induced RAW 264.7 cells. Additionally, the results of the CA-induced rat paw edema assay revealed an anti-edema effect of PS + CR. Furthermore, it is suggested that PS + CR also inhibits acute edematous inflammation by suppressing mast cell degranulation and inflammatory mediators (COX-2, iNOS, and TNF-α). Thus, PS + CR may be a potential candidate for the treatment of various inflammatory diseases, and it may also contribute to a better understanding of the molecular mechanisms underlying inflammatory response regulation.

## Abbreviations

CA: Carrageenan
COX: Cyclooxygenase
CR: *Coptis chinensis* root
DEXA: Dexamethasone
ELISA: Enzyme-linked immunesorbent assay
HE: Hematoxylin & Eosin
IL: Interleukin
iNOS: Inducible nitric oxide synthase
LPS: Lipopolysaccharide
MTT: 3-(4,5-dimethylthiazol-2-yl)-2,5-diphenyltetrazolium bromide
NF: Nuclear factor
NO: Nitric oxide
PBS: Phosphate-buffered saline
PG: Prostaglandin
p-I-κBα: Phospho-inhibitor of NF-κBα
PS: *Pelargonium sidoides*
RIPA: Radio immunoprecipitation assay
TNF: Tumor necrosis factor
